# Small interfering RNA (siRNA)-mediated knockdown of macrophage migration inhibitory factor (MIF) suppressed cyclin D1 expression and hepatocellular carcinoma cell proliferation

**DOI:** 10.18632/oncotarget.2141

**Published:** 2014-06-26

**Authors:** Xiao-hui Huang, Wei-hua Jian, Zhao-feng Wu, Jie Zhao, Hua Wang, Wen Li, Jin-tang Xia

**Affiliations:** ^1^ Laboratory of General Surgery, The First Affiliated Hospital, Sun Yat-Sen University, Guangzhou, Guangdong, China; ^2^ Department of General Surgery, The Third Affiliated Hospital, Guangzhou Medical University, Guangzhou, Guangdong, China; ^3^ Department of General Surgery, Guangzhou First Municipal People's Hospital, Guangzhou Medical University, Guangzhou, Guangdong, China

**Keywords:** Macrophage migration inhibitory factor, hepatocellular carcinoma, cyclin D1, RNA interference, proliferation

## Abstract

Macrophage migration inhibitory factor (MIF), a proinflammatory and immunoregulatory chemokine, plays important roles in cancer-related biological processes. However, few studies have focused on the clinical relevance of MIF and cyclin D1 expression in hepatocellular carcinoma cells (HCCs). In this study, MIF and cyclin D1 expression levels in HCC tissues and cell lines were significantly upregulated compared with adjacent normal tissues or a normal liver cell line. In HCC specimens, MIF expression positively correlated with cyclin D1 expression. Additionally, MIF and cyclin D1 expression positively correlated with tumor size. MIF knockdown inhibited the proliferation of PLC and HepG2 cells and promoted apoptosis. However, small interfering RNA (siRNA) against MIF did not influence the cell cycle in these cells. In an *in vivo* xenograft model, MIF knockdown reduced the tumor growth rate. The expression levels of Bcl-2, p-caspase-3, BIM and Bax were upregulated, while the expression levels of cyclin D1, p-Akt and p-ERK were downregulated in MIF-knockdown cells. These findings indicate that MIF siRNA reduces proliferation and increases apoptosis in HCC cells. MIF knockdown inhibits the expression of growth-related proteins and induces the expression of apoptosis-related proteins, supporting a role for MIF as a novel therapeutic target for HCC.

## INTRODUCTION

Hepatocellular carcinoma (HCC) is one of the most common cancers and the third leading cause of cancer-related deaths worldwide [1]. Dysregulated cellular proliferation caused by hepatitis-induced chronic inflammation is closely associated with HCC [2]. Although recent advances in surgical techniques and adjuvant treatments have improved survival, the incidence of recurrence remains high, and the long-term survival and prognosis for HCC patients is not satisfactory. Thus, finding new therapeutic strategies for HCC is critically important.

Numerous studies have shown that macrophage migration inhibitory factor (MIF) is a multi-functional cytokine associated with inflammation and tumorigenesis [3-4]. Recent studies have indicated that MIF is associated with HCC initiation and progression. Our previous study [5] demonstrated that the expression levels of serum MIF and VEGF were postively correlated in HCC patients, suggesting that MIF and VEGF play an important role in HCC progression. Yu et al reported [6] that MIF promotes cell proliferation and oncogenesis by maintaining the activity of extracellular signal-regulated kinase (ERK) and ERK2 mitogen-activated protein (MAP) kinases. Another recent study indicated that MIF regulates insulin/AKT signaling through angiotensin converting enzyme 2 (ACE2) [7]. The phosphoinositol-3-kinases (PI3K)/Akt pathway has been considered an important signaling pathway in the regulation of cell proliferation and differentiation [8]. Taken together, these results indicate that MIF may act as an upstream regulator of the AKT and ERK pathways.

The AKT and ERK signaling pathways promote the stabilization of cyclin D1 and regulate cyclin D1 expression [9-10]. Cyclin Dl is one of many cyclin proteins that regulate cell cycle progression from the G1 phase to the S phase [11]. Over-expression of cyclin D1 can lead to the dysregulation of cell proliferation and differentiation, thus promoting tumorigenesis [12]. However, there are few studies on the clinical relevance of MIF and cyclin D1 expression in HCC tissues and cells. Therefore, the aim of our study was to investigate the potential association between the expression of MIF and cyclin D1 with clinicopathologic parameters in HCC. To better understand the role of MIF and cyclin D1 in HCC cell growth, an additional aim of this study was to identity the effects of MIF knockdown on cell proliferation and apoptosis of PLC and HepG2 cells and cyclin D1 expression. Moreover, the activity of AKT, ERK and several apoptosis and proliferation-related proteins were also examined.

## RESULTS

### MIF and cyclin D1 expression and their clinicopathological significance in HCC

**Figure 1:**
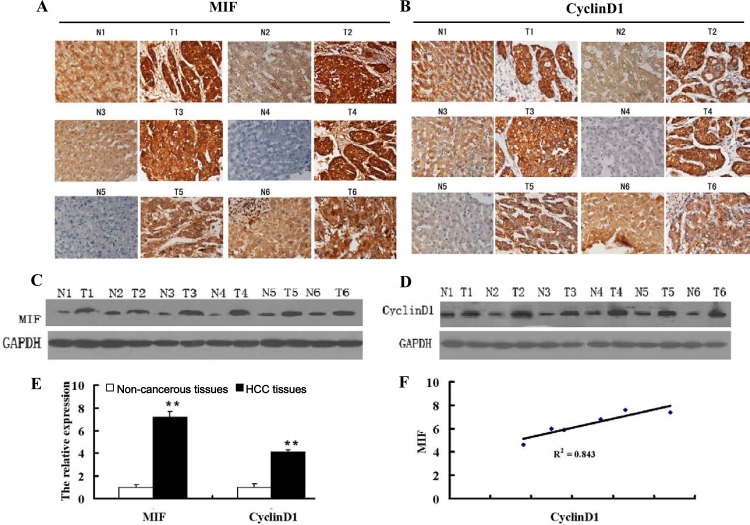
MIF and cyclin D1 are highly expressed in HCC (A) Immunohistochemical analysis of MIF expression in HCC tissue (T) and paired adjacent non-cancerous liver tissue (N) (200X magnification). (B) Immunohistochemical analysis of cyclin D1 expression in HCC tissue (T) and paired, adjacent non-cancerous liver tissue (N) (200X magnification). (C) MIF protein expression in HCC tissue (T) and paired, adjacent non-cancerous liver tissue (N). (D) Cyclin D1 protein expression in HCC tissue (T) and paired adjacent non-cancerous liver tissue (N). (E) MIF and cyclin D1 expression in HCC and paired adjacent non-cancerous tissue by qRT-PCR. The data were normalized to β-actin levels, as a control. (F) Correlation of MIF and cyclinD1 expression in HCC tissues. **P<0.001 compared with adjacent noncancerous liver tissues.

We evaluated the expression of MIF and cyclin D1 by performing immunohistochemistry on 93 tumor specimens from HCC patients. We found that MIF and cyclin D1 were overexpressed in 71% (66/93) and 41% (38/93) of tumor samples, respectively. The MIF and cyclin D1 proteins appeared to be located in the cytoplasm of tumor cells (Figure 1A, 1B). The adjacent non-cancerous tissues exhibited negative or weak MIF and cyclin D1 staining (Figure 1A, 1B). When investigating MIF and cyclin D1 expression with regard to HCC pathological factors, MIF expression was higher in large tumors (55/66) compared with small ones (11/66; Table 1). Cyclin D1 expression was also significantly higher in large tumors. Our results indicated that increased expression of MIF and cyclin D1 was significantly associated with tumor size. These results suggested that MIF expression, which correlated with cyclin D1 expression, was associated with HCC proliferation.

**Table 1:** Correlation between MIF and cyclinD1 expression and the clinicopathologic characteristics of the HCC patients

Variable feature	n	MIF		*P*	cyclinD1		*P*
		postive	negtive		postive	negtive	
Age				0.053			0.456
≥50	31	18	13		11	20	
<50	62	48	14		27	35	
Sex							
Male	78	57	21	0.477	35	43	0.477
Female	15	9	6		3	12	
Dfferention				0.145			0.145
well	2	2	3		1	4	
Presence	88	64	24		37	51	
Tumor size (cm)				0.005			0.007
≥3.5	70	55	15		30	40	
<3.5	23	11	12		8	15	
Metastasis				0.091			0.026
Yes	21	19	2		13	8	
No	72	47	25		25	47	
Hepatitis B infectuion				0.337			0.477
Presence	85	62	23		37	48	
Absence	8	4	4		1	7	
Serum AFP(μg/L)				0.841			0.125
≥20	60	43	17		28	32	
<20	33	23	10		10	23	

To confirm the expression of MIF and cyclin D1 in HCC tissues and adjacent non-cancerous tissues, real-time PCR and Western blot assays were performed on 6 pairs of HCC tissues and adjacent non-cancerous tissues. The results showed that mRNA and protein levels of MIF were higher in HCC tissues compared with the adjacent non-cancerous tissues (Figure 1C, 1E). Moreover, the mRNA and protein levels of cyclin D1 were also significantly higher in HCC tissues (Figure 1D, 1E), and MIF expression positively correlated with cyclin D1 expression (Figure 1F).

### Effect of MIF knockdown on MIF and cyclin D1 expression in HCC cells

**Figure 2:**
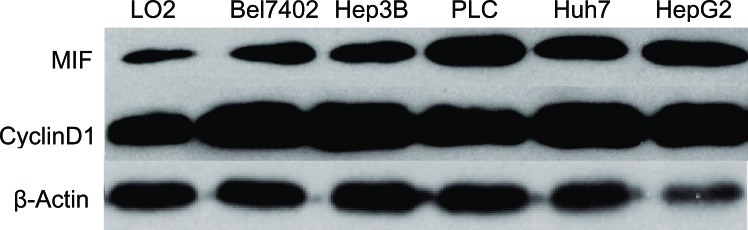
MIF and cyclin D1 expression is upregulated in HCC cell lines Western blot analysis of MIF and cyclin D1 expression in the human hepatocyte cell line LO2 and several HCC cell lines (BEL7402, PLC, HepG2, Huh-7 and Hep3B). β-Actin expression levels were used as internal controls.

To determine the expression levels of MIF and cyclin D1 in various HCC cell lines, we used real-time PCR and Western blot assays. MIF and cyclin D1 expression levels were evaluated in 5 HCC cell lines. Our results showed that MIF and cyclin D1 expression was upregulated in HCC cell lines compared with normal liver LO_2_ cells (Figure 2). The expression of MIF in PLC and HepG2 cells was substantially higher than other HCC cell lines, such as BEL7402, Hep3B and Huh7. Therefore, we used the PLC and HepG2 HCC cell lines as our cell model.

**Figure 3:**
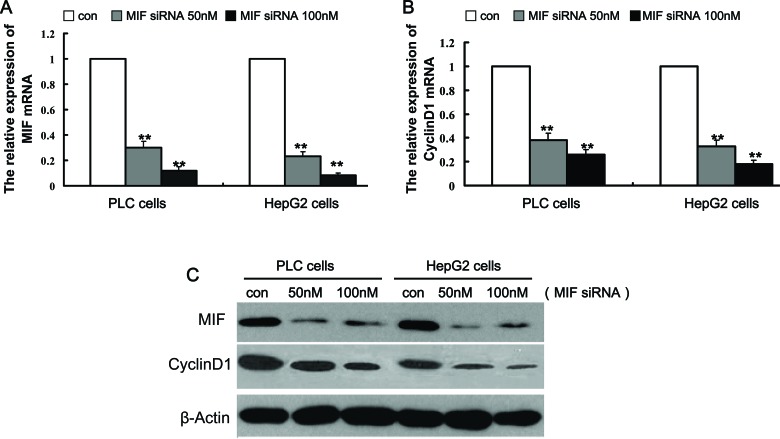
Knockdown of MIF expression by MIF siRNA.PLC and HepG2 cells were transfected with different concentrations of MIF siRNA or control siRNA for 48 h.RNA and protein expression was analyzed by real-time PCR and Western blot analysis, respectively (A) MIF and (B) cyclin D1 mRNA levels were downregulated by MIF siRNA. **P < 0.001 compared with controls. (C) Western blotting demonstrated that MIF protein and cyclin D1 protein levels were significantly decreased by MIF siRNA.

To knock down MIF expression in PLC and HepG2 cells, we designed an siRNA against MIF. The MIF siRNA was used at different concentrations to transfect PLC and HepG2 cells, and real-time PCR and Western blotting analyses were performed to measure MIF expression. The results showed that 50 and 100 nM MIF siRNA effectively decreased MIF expression by 78% and 92%, respectively (Figure 3A, 3C). Therefore, 50 and 100 nM MIF siRNA were used in all subsequent experiments. Meanwhile, 50 and 100 nM MIF siRNA inhibited cyclin D1 expression levels by 72% and 78%, respectively (Figure 3B, 3C).

### Effect of MIF knockdown on HCC cell proliferation

**Figure 4:**
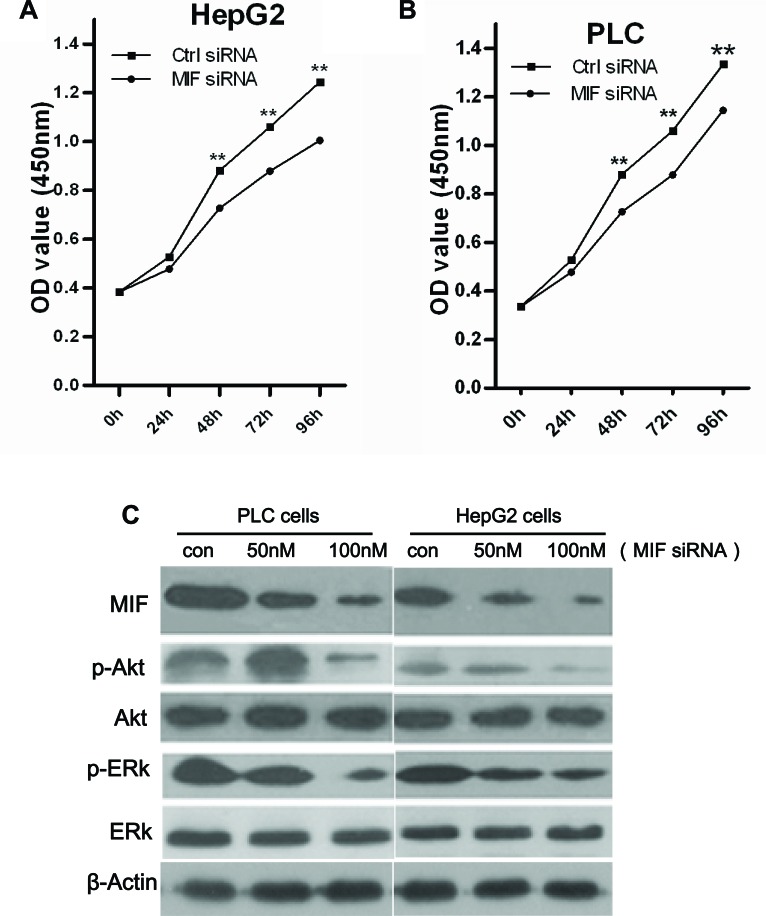
The effects of MIF knockdown on cell proliferation (A) HepG2 cells and (B) PLC cells were transfected with MIF or control siRNA. Cell viability was assessed using MTT assays at five time points (0, 24, 48, 72, and 96 h). **P < 0.01. (C) Western blot analysis showed that knockdown of MIF expression reduced the expression of p-AKT and p-ERK in PLC and HepG2 cells.

To examine the effects of MIF knockdown on cellular proliferation, PLC and HepG2 cells were transfected with 100 nM MIF siRNA for 24, 48, 72 or 96 h. MTT assays were performed to evaluate cell proliferation. As shown in Figure 4A and 4B, transfection of MIF siRNA inhibited proliferation in a time-dependent manner in PLC and HepG2 cells (P < 0.05). These data indicate that suppression of MIF expression is sufficient to inhibit PLC and HepG2 cell proliferation.

To investigate the underlying mechanisms by which MIF regulates proliferation of hepatic cancer cells, real-time PCR and Western blots were performed to determine the expression of proliferation-related genes. Compared with the cells transfected with control siRNA, the expression levels of cyclin D1 were significantly decreased in HCC cells (Figure 3C). In addition, the levels of p-AKT and p-ERK were significantly decreased after MIF knockdown. However, there was no significant difference in the expression levels of total AKT and ERK (Figure 4C).

### Effect of MIF knockdown on cell cycle regulation and apoptosis in HCC

**Figure 5:**
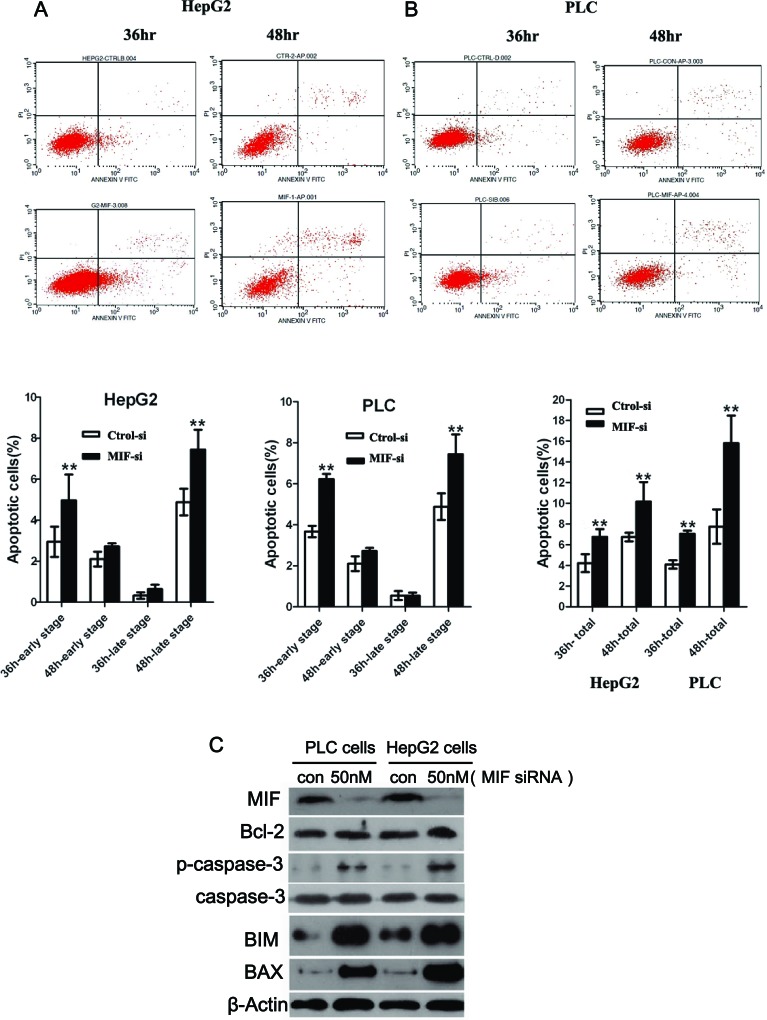
The effects of MIF knockdown on cell apoptosis (A) HepG2 cells and (B) PLC cells were transfected with MIF or control siRNA. Apoptosis was assessed using the Annexin V-FITC Apoptosis Detection Kit. **P < 0.01. (C) Western blot analysis showed that MIF knockdown increased the expression of Bcl-2, p-caspase-3, BIM and BAX in PLC and HepG2 cells. **P < 0.01.

The effects of MIF knockdown on PLC and HepG2 cell cycle progression and apoptosis were analyzed by flow cytometry. The Fluorescence Activated Cell Sorter (FACS) results indicated that MIF knockdown did not have a significant effect on the cell cycle (Supplementary Figure 1). However, MIF siRNA resulted in a significant increase in apoptosis compared with untreated cells; MIF knockdown significantly promoted late stage apoptosis (Figure 5A, 5B).

To further evaluate the mechanism of apoptosis induced by MIF knockdown, we measured the expression of apoptosis-related signaling proteins. PLC and HepG2 cells were treated with MIF siRNA for 24 h; then, the cell lysates were immunoblotted with Bcl-2, p-caspase-3, caspase-3 and BIM antibodies. The expression levels of Bcl-2, p-caspase-3 and BIM were significantly upregulated in the MIF siRNA groups (Figure 5C), while the expression of caspase-3 remained unchanged irrespective of the MIF siRNA treatment (Figure 5C).

### Effect of MIF knockdown on HCC tumorigenicity in a xenograft model

**Figure 6:**
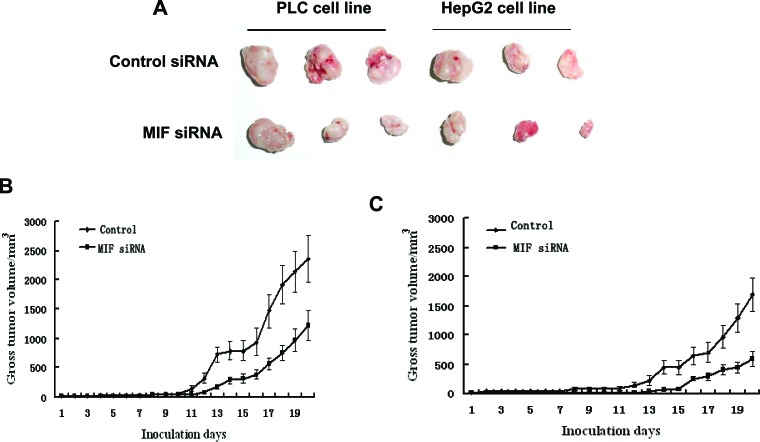
The effects of MIF knockdown on PLC and HepG2 cell growth in nude mice (A) Images of tumors extracted from MIF-knockdown and control groups (PLC and HepG2) after nude mice were euthanized 21 d post-tumor cell injection. (B) Growth curves for PLC tumors treated with MIF or control siRNA. (C) Growth curves for HepG2 tumors treated with MIF or control siRNA.

In this study, we examined the tumorigenicity of HCC cells treated with MIF siRNA *in vivo*. Our *in vitro* data showed that MIF knockdown by siRNA significantly reduced cell proliferation. Therefore, we further investigated the effect of MIF siRNA on a human HCC xenograft *in vivo*. To determine the effect of MIF siRNA on tumor growth, mice were treated with control or MIF siRNA and observed for 21 d. A representative image shows reduced tumor growth in the MIF siRNA-treated group compared with the control siRNA-treated group (Figure 6A). The tumor volume of mice injected with MIF siRNA showed significantly reduced tumor growth compared with the mice administered control siRNA (Figure 6B, 6C). These results suggest that MIF may be a molecular target for novel cancer treatment modalities.

## DISCUSSION

Although a variety of MIF functions have been reported, the precise role of MIF in HCC progression remains unknown. We previously reported [5] that serum MIF concentrations are higher in HCC patients than in controls. MIF overexpression was significantly associated with tumor size and intrahepatic metastasis, suggesting that MIF plays an important role in HCC progression. It is generally acknowledged that the activation of the ERK and AKT signaling pathways leads to aggravated effects in HCC [13-14]. Rie et al reported [15] that a decrease in cyclin D1 expression, owing to inhibition of the MAPK and AKT pathways, might be a mechanism by which HSP20 controls HCC proliferation. MIF may act as an upstream regulator of the AKT and ERK pathways. Therefore, we hypothesized that MIF is associated with cyclin D1, and the present study focused on the clinical relevance of MIF and cyclin D1 expression in HCC tissues and cells. In addition, MIF knockdown may influence the expression of cyclin D1 and the proliferative capacity of HCC cell lines.

In this study, the expression of MIF in PLC and HepG2 cell lines was substantially higher than other HCC cell lines (BEL7402, Hep3B, and Huh7). Gerber reported that the enzyme phenotype of PLC cells displays remarkable resemblance to those observed in human HCC [16]. The HepG2 cell line was established from liver tumor biopsies obtained during extended lobectomies of a 15-year-old Caucasian male from Argentina [17]. The HepG2 cell line retains many of the specialized functions normally lost by primary hepatocytes in culture such as secretion of the major plasma proteins [18]. Therefore, we speculated that the expression of MIF in PLC and HepG2 cells would be higher than other HCC cell lines owing to their endogenous expression of MIF. In addition, MIF expression positively correlated with cyclin D1 expression in HCC tissues and was closely associated with HCC tumor size. These results suggest that overexpression of both MIF and cyclin D1 is involved in regulating HCC proliferation. Consistent with our results, MIF is overexpressed in other tumors, including glioblastoma [19] and breast cancer [20]. A study by James D et al suggested that MIF-mediated Rho activity promotes steady-state cyclin D1 expression and Rb inactivation in rodent fibroblasts [21].

To further investigate the pathobiological role of MIF in the proliferation of HCC, we introduced MIF-specific siRNA constructs into PLC and HepG2 cells and assessed the effects on cyclin D1 expression, cell proliferation, cell cycle and cell apoptosis. MIF siRNA inhibited cell proliferation and decreased cyclin D1 expression, while inducing cell apoptosis. These results suggest that MIF plays an important role in HCC cell proliferation and apoptosis. However, our results showed that MIF siRNA did not cause significant changes in the cell cycle in either PLC or HepG2 cells compared with control, and the reason remains unclear.

MIF is known to influence the signaling of growth and apoptosis-regulating pathways [22]. The activation of signaling pathways, such as the ERK or AKT pathways, predicts poor patient prognosis and early recurrence of HCC [23]. Therefore, we examined the phosphorylation levels of ERK and AKT. Our results showed that the phosphorylation levels of ERK and AKT were dramatically lower in MIF-siRNA cells than in control cells. Similar observations have been made using siRNA targeting of MIF in prostate cancer [24] and ovarian cancer cells [25]. Moreover, we showed that cyclin D1 expression could be downregulated by MIF siRNA. Cyclin D1 is a major regulator of cell cycle progression [26]. Gene amplification and abnormal expression of cyclin D1 have been described in several human cancers [27-28]. Some research has demonstrated that MIF binds to the surface receptors CD74 and CXCR2/CXCR4, thereby stimulating signaling pathways, such as MAPK, NF-κB, and AKT [29-31]. MIF induced sustained ERK activation, leading to an increase in cyclin D1 expression [21]. Therefore, our studies suggest that MIF siRNA may suppress the ERK/AKT-cyclin D1 pathway to inhibit HCC cell proliferation.

MIF siRNA also significantly promoted apoptosis of PLC and HepG2 cells, increasing the percentage of apoptotic cells. Tumor development requires decreased apoptosis of cancerous cells. Therefore, increasing apoptosis is a promising strategy for suppressing tumor progression [32-33]. To assess whether inhibition of HCC cell growth by MIF knockdown correlates with the induction of apoptosis, the levels of apoptosis-related proteins were assessed. The results indicated that MIF siRNA significantly increased BAX, Bcl-2, BIM and p-caspase-3 protein levels, as determined by Western blot analysis. Cytokines may directly trigger mitochondria-associated pro-apoptotic proteins that depend largely upon the action of BAX and caspase 3 [34]. Our results suggest that MIF siRNA impairs the activation of both BAX production and caspase 3. Therefore, our studies suggest that MIF siRNA facilitates apoptosis of HCC cells through the mitochondria-dependent pathway.

Additionally, to investigate the function of MIF siRNA in HCC *in vivo*, siRNA-transfected and parental PLC and HepG2 cells were orthotopically transplanted into nude mice. The results revealed that MIF siRNA significantly suppressed tumor growth in nude mice. Consistent with our finding, a recent report showed that MIF knockdown inhibited the tumorigenicity of breast cancer cells in a tumor xenograft mouse model [35].

The present study demonstrates that MIF may be a potential tumor biomarker and therapeutic target because it is overexpressed in human HCC. Additionally, MIF may interact with cyclin D to promote HCC tumor growth. When MIF expression was silenced, cyclin D1 protein and mRNA levels in cells were also reduced. The biological mechanism by which MIF acts to promote HCC cell growth includes the induction of growth-related protein expression and inhibition of apoptosis-related protein expression. This mechanism may explain why MIF knockdown led to an inhibition of cell proliferation and an increase in apoptosis.

In conclusion, our study demonstrates that MIF and cyclin D1 are overexpressed in HCC tissues and cells. MIF siRNA significantly inhibited HCC proliferation and induced apoptosis, and the mechanism by which this occurs involves MIF siRNA-induced inhibition of growth-related protein expression and an induction of apoptosis-related protein expression. Although additional functional studies are required, our results suggest that suppression of MIF by RNA interference may be a potentially important therapeutic modality for MIF-positive cancers.

## MATERIALS AND METHODS

### Tissue samples and reagents

Fresh tumor specimens were obtained from patients who underwent resection of primary HCC in the Department of Hepatobiliary Surgery at the First Affiliated Hospital of Sun Yat-Sen University. Representative blocks from both tumor (T) and tumor adjacent non-cancerous tissues (N) from each specimen were stored at -80°C for RNA and protein extraction. All human tissue specimens were obtained after obtaining patient's consent and approval from the Institute Research Ethics Committee of the First Affiliated Hospital of Sun Yat-Sen University. None of the patients had previously received chemotherapy or radiation therapy.

### Cell culture

The PLC, HepG2 and Hep3B cell lines were obtained commercially from American Type Culture Collection (ATCC). The LO2 and BEL-7402 cell lines were purchased from the Institute of Biochemistry and Cell Biology in Shanghai, China. All of the cells were cultured in DMEM supplemented with 10% (v/v) heat inactivated FBS, 100 units/ml penicillin and 100 μg/ml streptomycin in a humidified 5% CO_2_ incubator at 37°C. Tissue culture flasks were purchased from Corning (Corning, NY).

### MIF siRNA treatment

Double-stranded siRNAs (dsRNA) targeting the MIF gene (NM-002415) and complementary dsRNA were synthesized by Chemical Methods (ReiBo Biotech, China). The nucleotide sequence of the dsRNA for human MIF mRNA was 5'-ggg ucu aca uca acu auu a.dTdT-3'and 3'-dTdTccc aga ugu agu uga uaa u-5'. The nucleotide sequence of the control siRNA from a scramble sequence was 5'-uuc ucc gaa cgu guc acg u.dTdT-3' and dTdT aag agg cuu gca cagugc a-5'. Cells were seeded at 5×10^5^ cells per well in 6-well plates in DMEM containing 10% FBS without penicillin and streptomycin overnight. Transfection experiments were performed with OPTI-MEM serum-free medium and Lipofectamine 2000 reagent with a final siRNA concentration of 50 or 100 nM. The cells were collected for MTT assay and RNA and protein extraction after 24 h of siRNA transfection.

### Real-time reverse transcription-polymerase chain reaction (RT-PCR)

Total RNA from cells and tumors was extracted using TRIzol reagent (Invitrogen, Carlsbad, CA, USA) according to the manufacturer's protocol. RNA concentration and quality were assessed spectrophotometrically at 260 and 280 nm. The enzyme and reagents for reverse transcription and PCR amplification were obtained from Invitrogen.

MIF and cyclin D1 mRNA levels were measured by quantitative PCR amplification. After siRNA treatment in PLC and HepG2 cells using EvaGreen dye (Molecular Probes, Inc., USA), mRNA levels were assessed using an ABI 7500 PCR machine. β-Actin PCR products were used as a loading control. Amplification (40 cycles) was performed in a total volume of 25 μL containing dNTP mix, blend Taq Plus polymerase, 10× blend Taq Plus buffer, 20× EvaGreen dye, 50× ROX Reference Dye (Invitrogen, USA), oligo-dT (100 pmol/L) and 0.75 μL each of the sense and antisense primer-specific nucleotide sequences for MIF, cyclin D1, and β-actin, as described in Table 2. Melt curve analysis was performed at the end of each PCR reaction. The mRNA expression levels of MIF and cyclin D1 were quantified and expressed relative to β-actin expression. The data were analyzed using the 2^-ΔΔCt^ method, which presents the data as changes in gene expression relative to β-actin. RQ ≤ 0.5 was defined as a significant down regulation of gene expression. RQ ≥ 2 was defined as a significant up regulation of gene expression.

**Table 2:** Real-time PCR primers for amplification of MIF, cyclin D1 and β-actin

Gene	base number	sense primer(5'-3')	antisense primer(5'-3')
MIF	141bp	GCAGAACCGCTCCTACAGCA	GGCTCTTAGGCGAAGGTGGA
CyclinD1	146bp	GCTGCTCCTGGTGAACAAGC	CACAGAGGGCAACGAAGGTC
β-actin	144bp	CCTGGCACCCAGCACAAT	GGGCCGGACTCGTCATACT

MIF and cyclin D1 mRNA expression levels in tumor tissues (T) and tumor adjacent non-cancerous tissues (N) from each HCC specimen were measured with semi-quantitative RT-PCR. The primers used for amplification were the same as those used in Table 1. β-Actin expression was analyzed as a control. The cDNAs were amplified by PCR (94°C for 30 s, 55°C for 30 s, and 72°C for 30 s) for 30 cycles and a final extension at 72°C for 7 minutes using a thermal cycler (ABI 9700, Applied Biosystems). The PCR products were electrophoresed on a 1.5% agarose gel. The mRNA expression levels of MIF and cyclin D1 were quantified relative to β-actin expression. The results are presented as a fold increase in mRNA expression relative to the amount present in tumor adjacent non-cancerous tissues (N). Each experiment was performed in triplicate wells in three independent experiments.

### Western blot analysis

Cells were washed three times with cold phosphate-buffered saline (PBS), and directly lysed in RIPA-buffer (0.5% sodium deoxycholate, 0.1% SDS, 1% NP-40 and PBS) with PMSF and proteases inhibitors. Tissue samples were ground into a powder with liquid nitrogen and lysed in RIPA buffer. The amount of whole cell proteins was adjusted for equal loading (30 micrograms per lane). Next, the proteins were separated on a 10% SDS-polyacrylamide gel electrophoresis (SDS-PAGE) gel and transferred onto PVDF membranes (Millipore Corporation, Bedford, MA, USA). The PVDF membrane was then blocked with 10% skim milk in Tris-buffered saline containing 0.1% Tween 20 (TBST) for one hour and incubated with a rabbit polyclonal anti-human MIF antibody or anti-human cyclin D1 monoclonal antibody (Santa Cruz biotechnology, Inc.) at a 1:1000 dilution overnight at 4°C. Rabbit anti-human phospho-p44/p42 MAPK or total p44/p42 MAPK primary antibodies (Cell Signaling Technology, Beverly, MA, USA) at a 1:1000 dilution were incubated for 2 h at room temperature. The peroxidase-conjugated anti-mouse or anti-rabbit IgG and HRP-conjugated monoclonal mouse anti-glyceraldehyde-3-phosphate dehydrogenase (GAPDH) antibodies were incubated for 1 h at room temperature. The immunoreactive bands were visualized with an ECL chemiluminescence system (Pierce, Rockford, USA) and analyzed with the Gene Tools V3.04b software. All experiments were repeated at least three times with similar results.

### 3-(4, 5-Dimethylthiazol-2-yl)-2, 5-diphenyltetrazolium bromide (MTT) assay

Cell proliferation was examined using the 3-(4, 5-dimethylthiazol-2-yl)-2, 5-diphenyltetrazolium bromide (MTT) colorimetric assay according to the manufacturer's protocol (Cell Proliferation Kit, Roche). After 24 h of treatment with MIF or control siRNA (100 nM), PLC and HepG2 cells were trypsinized and placed in a 96-well plate with 5,000 cells per well for an additional 48 h. Absorbance at 570 nm was measured using a microtiter plate reader (Multiskan MK3, Thermo Electron Corporation, USA). Data were obtained from three separate experiments. The percentage of decrease in proliferation in the MIF siRNA groups was determined by comparing the absorbance values in the siRNA controls. Each experiment was repeated at least three times in quadruplicate wells.

### Immunohistochemistry staining

Immunohistochemistry (IHC) was performed on 93 formalin-fixed, paraffin-embedded primary HCC tumors that were histopathologically and clinically diagnosed at the Department of Pathology and the Department of Hepatobiliary Surgery, First Affiliated Hospital, SUN Yat-Sen University between 1997 and 2001 using the Dako EnVision™ method. Tissue sections were deparaffinized, rehydrated and microwave-treated in a 10 mM citrate buffer (pH 6.0) for 10 min for antigen retrieval. Endogenous peroxidase activity was blocked with 0.3% hydrogen peroxide for 15 min. Sections were incubated with anti-human MIF (1:200 dilution) or anti-cyclin D1 (1:100 dilution) at room temperature for 30 min. Next, tissue sections were incubated with ready-to-use HRP-immunoglobulin (EnVision™) for 30 min and developed using 3'-3' diaminobenzidine (DAB) as a chromogen substrate.

### Apoptosis assay

At 36 or 48 h after transfection, the cells were harvested and washed twice with cold PBS. Next, the cells were stained with Annexin V-FITC and 10 μl propidium iodide (PI) using the Annexin V-FITC Apoptosis Detection Kit (KEYGEN, Nanjing, China). The percentage of apoptotic cells was detected using a FACSCalibur Flow cytometer (BD, San Jose, CA, USA). All analyses were performed in triplicate.

### Cell cycle analysis

Cells transfected with MIF siRNA or control siRNA were collected 24 h after transfection. The cells were fixed with cold 75% ethanol at 4°C overnight, washed in cold PBS, and stained with propidium iodide (50 ng) containing RNase (100 ng) for 30 min at 37°C. The percentage of cells in different phases of the cell cycle was measured by flow cytometry (BD, San Jose, CA, USA). The results were analyzed using the FlowJo 2.8 software. The experiment was performed in triplicate.

### Tumor growth in nude mice

BALB/c nude mice (5 week old, male, n = 18) were purchased from Guangzhou University of Chinese Medicine (Guangzhou, China) and maintained under pathogen-free conditions in the Ophthalmic Animal Laboratory of Zhong Shan Ophthalmic Center, Sun Yat-Sen University (Guangzhou, China). All procedures followed the experimental protocols approved by the animal care committee of Sun Yat-Sen University. The mice were injected with either PLC or HepG2 cells and divided into two groups: the control groups were treated with control siRNA (100 nM); and the MIF-siRNA group was treated with MIF siRNA (100 nM). In total, 5 × 10^6^ PLC or HepG2 cells resuspended in PBS were subcutaneously injected into left flank of the mice. Tumor growth in each group was monitored by taking 2-dimensional measurements of tumors from each mouse every day between days 3 and 21 following tumor inoculation. All of the mice were sacrificed on day 21, and the tumor nodules were weighed.

### Statistical analyses

All data were expressed as the mean ± SD (standard deviation) and compared using ANOVA. The χ^2^ test was used for comparisons between immunohistochemical and clinicopathological parameters. Spearman's bivariate correlation test was used to evaluate correlations between MIF and cyclin D1 expression. Statistical significance was assumed as *p* < 0.05. All statistical analyses were performed using the SPSS 13.0 statistical software.

## SUPPLEMENTARY MATERIAL AND FIGURE


