# Supplementary material for: Small interfering RNA (siRNA)-mediated knockdown of macrophage migration inhibitory factor (MIF) suppressed cyclin D1 expression and hepatocellular carcinoma cell proliferation
Source: Oncotarget. 2014 Jun 26;5(14):5570–80. doi: 10.18632/oncotarget.2141 (PMC4170598; doi:10.18632/oncotarget.2141)
Supplement: Supplementary file 1 [file oncotarget-05-5570-s001.pdf]

# Small interfering RNA (siRNA)-mediated knockdown of macrophage migration inhibitory factor (MIF) suppressed cyclin D1 expression and hepatocellular carcinoma cell proliferation

**Supplementary Material**

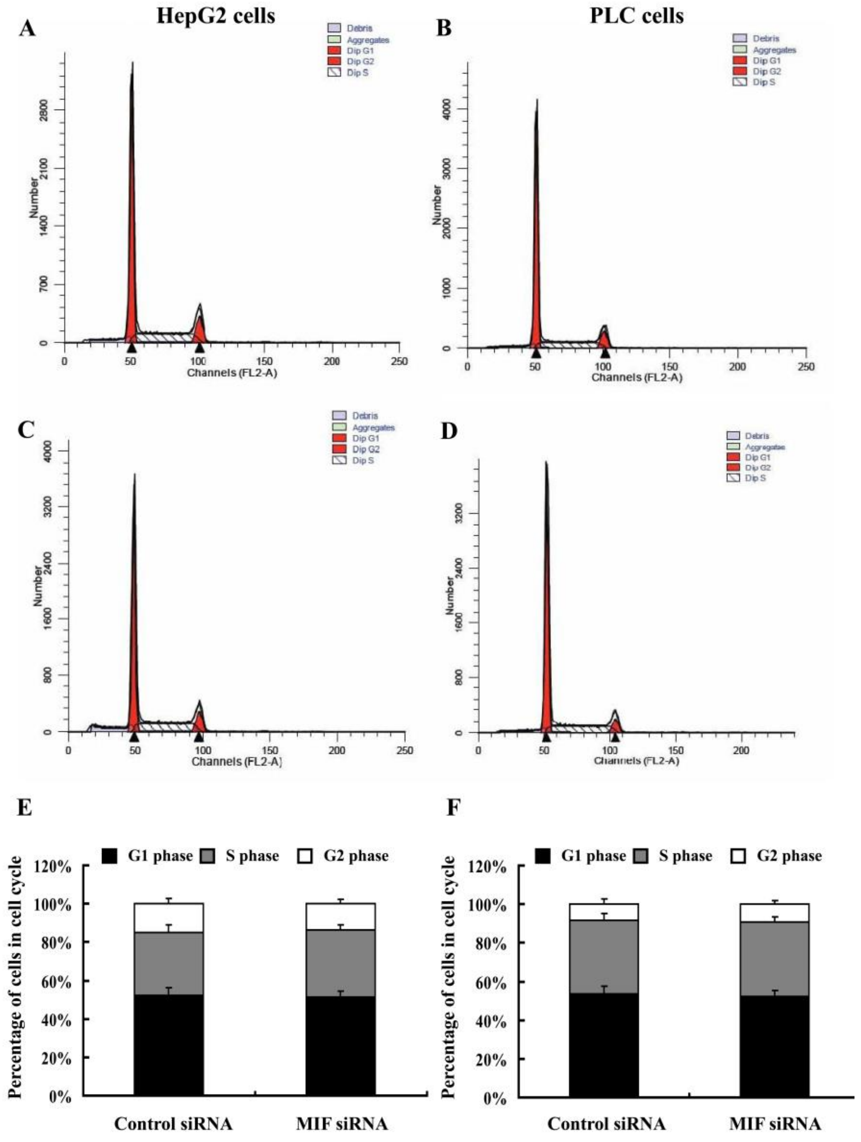


**Supplementary Figure 1: MIF knockdown did not affect the cell cycle in PLC and HepG2 cells.** (A) The cell cycle phases were detected in HepG2 cells treated with control siRNA by flow cytometry. (B) The cell cycle phases were detected in PLC cells treated with control siRNA by flow cytometry. (C) The cell cycle phases were detected in HepG2 cells treated with MIF siRNA by flow cytometry. (D) The cell cycle phases were detected in PLC cells treated with MIF siRNA by flow cytometry. (E) The data are described for the cell cycle of HepG2 cells treated with control or MIF siRNA. (F) The data are described for the cell cycle of PLC cells treated with control or MIF siRNA.
